# Diversity of A(H5N1) clade 2.3.2.1c avian influenza viruses with evidence of reassortment in Cambodia, 2014-2016

**DOI:** 10.1371/journal.pone.0226108

**Published:** 2019-12-09

**Authors:** Annika Suttie, Songha Tok, Sokhoun Yann, Ponnarath Keo, Srey Viseth Horm, Merryn Roe, Matthew Kaye, San Sorn, Davun Holl, Sothyra Tum, Philippe Buchy, Ian Barr, Aeron Hurt, Andrew R. Greenhill, Erik A. Karlsson, Dhanasekaran Vijaykrishna, Yi-Mo Deng, Philippe Dussart, Paul F. Horwood

**Affiliations:** 1 Virology Unit, Institut Pasteur du Cambodge, Institut Pasteur International Network, Phnom Penh, Cambodia; 2 School of Health and Life Sciences, Federation University, Churchill, Australia; 3 WHO Collaborating Centre for Reference and Research on Influenza, Victorian Infectious Diseases Reference Laboratory, at the Peter Doherty Institute for Infection and Immunity, Melbourne, Victoria, Australia; 4 National Animal Health and Production Research Institute, General Directorate of Animal Health and Production, Cambodian Ministry of Agriculture, Forestry and Fisheries, Phnom Penh, Cambodia; 5 GlaxoSmithKline Vaccines R&D Intercontinental, Singapore, Singapore; 6 Department of Microbiology, Biomedicine Discovery Institute, Monash University, Melbourne, Victoria, Australia; 7 College of Public Health, Medical and Veterinary Sciences, James Cook University, Townsville, Queensland, Australia; St. Jude Children’s Research Hospital, UNITED STATES

## Abstract

In Cambodia, highly pathogenic avian influenza A(H5N1) subtype viruses circulate endemically causing poultry outbreaks and zoonotic human cases. To investigate the genomic diversity and development of endemicity of the predominantly circulating clade 2.3.2.1c A(H5N1) viruses, we characterised 68 AIVs detected in poultry, the environment and from a single human A(H5N1) case from January 2014 to December 2016. Full genomes were generated for 42 A(H5N1) viruses. Phylogenetic analysis shows that five clade 2.3.2.1c genotypes, designated KH1 to KH5, were circulating in Cambodia during this period. The genotypes arose through multiple reassortment events with the neuraminidase (NA) and internal genes belonging to H5N1 clade 2.3.2.1a, clade 2.3.2.1b or A(H9N2) lineages. Phylogenies suggest that the Cambodian AIVs were derived from viruses circulating between Cambodian and Vietnamese poultry. Molecular analyses show that these viruses contained the hemagglutinin (HA) gene substitutions D94N, S133A, S155N, T156A, T188I and K189R known to increase binding to the human-type α2,6-linked sialic acid receptors. Two A(H5N1) viruses displayed the M2 gene S31N or A30T substitutions indicative of adamantane resistance, however, susceptibility testing towards neuraminidase inhibitors (oseltamivir, zanamivir, lananmivir and peramivir) of a subset of thirty clade 2.3.2.1c viruses showed susceptibility to all four drugs. This study shows that A(H5N1) viruses continue to reassort with other A(H5N1) and A(H9N2) viruses that are endemic in the region, highlighting the risk of introduction and emergence of novel A(H5N1) genotypes in Cambodia.

## Introduction

Avian influenza viruses (AIVs; family *Orthomyxoviridae*) continuously circulate globally in their natural reservoir, wild waterbirds (Orders *Anseriformes* and *Charadriiformes*). Frequent spill over and establishment in domestic poultry species increases risk of zoonotic infection. The majority of AIVs circulate as low pathogenic avian influenza viruses that cause mild or no disease in domestic poultry [1,2]. However, subtype A(H5) and A(H7) AIVs are also capable of mutating to form highly pathogenic avian influenza (HPAI) viruses that can cause high morbidity and mortality in poultry flocks [3,4]. The first outbreak of A(H5N1) HPAI viruses occurred among geese in Guangdong, China during 1996 [5]. Since that time, the A/goose/Guangdong/1/1996 (Gs/Gd) A(H5N1) lineage has disseminated globally causing intermittent outbreaks in domestic poultry and sporadic human infections [6,7]. As of September 2019, 861 confirmed human cases of A(H5N1) infection, resulting in 455 fatalities (case fatality rate, CFR: 53%) have been reported from 17 countries [8]. The primary risk factor for human infection with AIVs is close contact to poultry [9,10] and *in vivo* studies have shown that A(H5) viruses (with as few as five amino acid substitutions) can acquire aerosol transmissibility in ferrets [11,12]. Fortunately, sustained transmission of A(H5) AIVs between humans has not been documented, though mutations enabling greater transmissibility among humans greatly increases the pandemic threat [13,14].

Influenza A viruses consist of eight negative sense single-stranded RNA segments, each encoding one or more viral proteins. Influenza A viruses are subtyped based on the hemagglutinin (HA) and neuraminidase (NA) glycoproteins that are present on the surface of the viral envelope. There have been eighteen HA subtypes (H1-H18) and eleven NA subtypes (N1-N11) identified. Subtypes H1-H16 and N1-N9 have mainly been identified in avian species, whereas H17-H18 and N10-N11 have only been detected in bats. The HA protein, which is responsible for initiating viral infection by binding to sialic acid receptors on the surface of host cells, is particularly important in host restriction and pathogenicity. The HAs of AIVs preferentially bind to alpha 2,3 (α2,3)-linked sialic acid receptors found in the intestinal and respiratory tract of avian species [15]. While human seasonal influenza viruses preferentially bind to α2,6-liked sialic acid receptors predominantly found in the human upper respiratory tract. Amino acid substitutions in the receptor binding pocket of the HA gene, such as Q222L and G224S (H5 numbering), have been associated with a switch in receptor binding preference from avian-type α2,3 to human-type α2,6 receptors [16]. The acquisition of α2,6 specificity is of concern as it increases AIV transmissibility in mammalian species, increasing their pandemic potential [11,12].

AIVs are a major concern in Southeast Asia. In Cambodia, AIV outbreaks can have devastating socioeconomic consequences as a large proportion of Cambodians rely on agriculture for their livelihoods [17]. A(H5N1) HPAI viruses were first detected in Cambodia during an AIV outbreak in poultry during January 2004 [6]. Since then, A(H5N1) has become endemic in the country, resulting in 56 human infections with 37 deaths (CFR 66%, June 2019) [18]. AIV surveillance in Cambodian poultry has been conducted since 2006 and consists of active surveillance in prominent live bird markets (LBMs) and passive detection following investigations of disease outbreaks in poultry. Human monitoring for zoonotic AIVs consists of a country-wide influenza-like-illness sentinel system, severe acute respiratory illness and event-based surveillance [19,20].

Based on the A(H5) HA clade nomenclature described by the WHO/OIE/FAO H5 Evolution Working Group [21], all Cambodian A(H5N1) viruses identified prior to 2014 belonged to clade 1, or its associated subclades (1.1, 1.1.1 and 1.1.2) [22]. In 2013, a reassortant clade 1.1.2 A(H5N1) virus emerged in Cambodia. The HA and NA genes were from clade 1.1.2, whereas the MP and all internal genes clustered with clade 2.3.2.1a [21–23]. This reassortant caused numerous outbreaks in poultry and was associated with a dramatic increase in human cases during 2013 (n = 26) [23] and early 2014 (n = 8) [24]. The reassortant virus was subsequently replaced by a clade 2.3.2.1c virus after March 2014. Interestingly, only one human case has been detected since this time. Here, we seek to understand the diversity and molecular evolution of A(H5N1) clade 2.3.2.1c viruses in Cambodia, particularly in regard to human and avian disease risk. This study reports the genetic diversity and molecular evolution of Cambodian A(H5N1) clade 2.3.2.1c viruses detected from 2014 to 2016 through routine surveillance in LBMs, from poultry outbreak investigations and human cases.

## Materials and methods

### Ethical approval

Animal sampling was conducted by the National Animal Health and Production Research Institute (NAHPRI) under the direction of the General Directorate for Animal Health and Production, Cambodian Ministry of Agriculture, Forestry and Fisheries as part of routine disease surveillance activities; thus, poultry sampling was not considered as experimental animal research. The analysis of poultry samples for avian influenza testing was approved by the Cambodian National Ethics Committee for Health Research (approval #051NECHR). The Institut Pasteur du Cambodge (IPC) serves as a World Health Organization H5 Reference Laboratory and the Cambodian National Influenza Center, with approvals and infrastructure necessary to work on highly pathogenic avian influenza. No animal experimentation was performed at IPC.

### Sample collection

Samples included in this analysis were collected as part of passive and active surveillance systems used to monitor AIV circulation in Cambodia from 2014 to 2016. Surveillance efforts in poultry were co-ordinated by the Virology Unit at IPC and NAHPRI under the direction of the General Directorate for Animal Health and Production, Cambodian Ministry of Agriculture, Forestry and Fisheries. Poultry outbreaks of influenza A(H5N1) were investigated by NAHPRI and positive samples were sent to IPC for confirmation and viral characterization.

**Fig 1:**
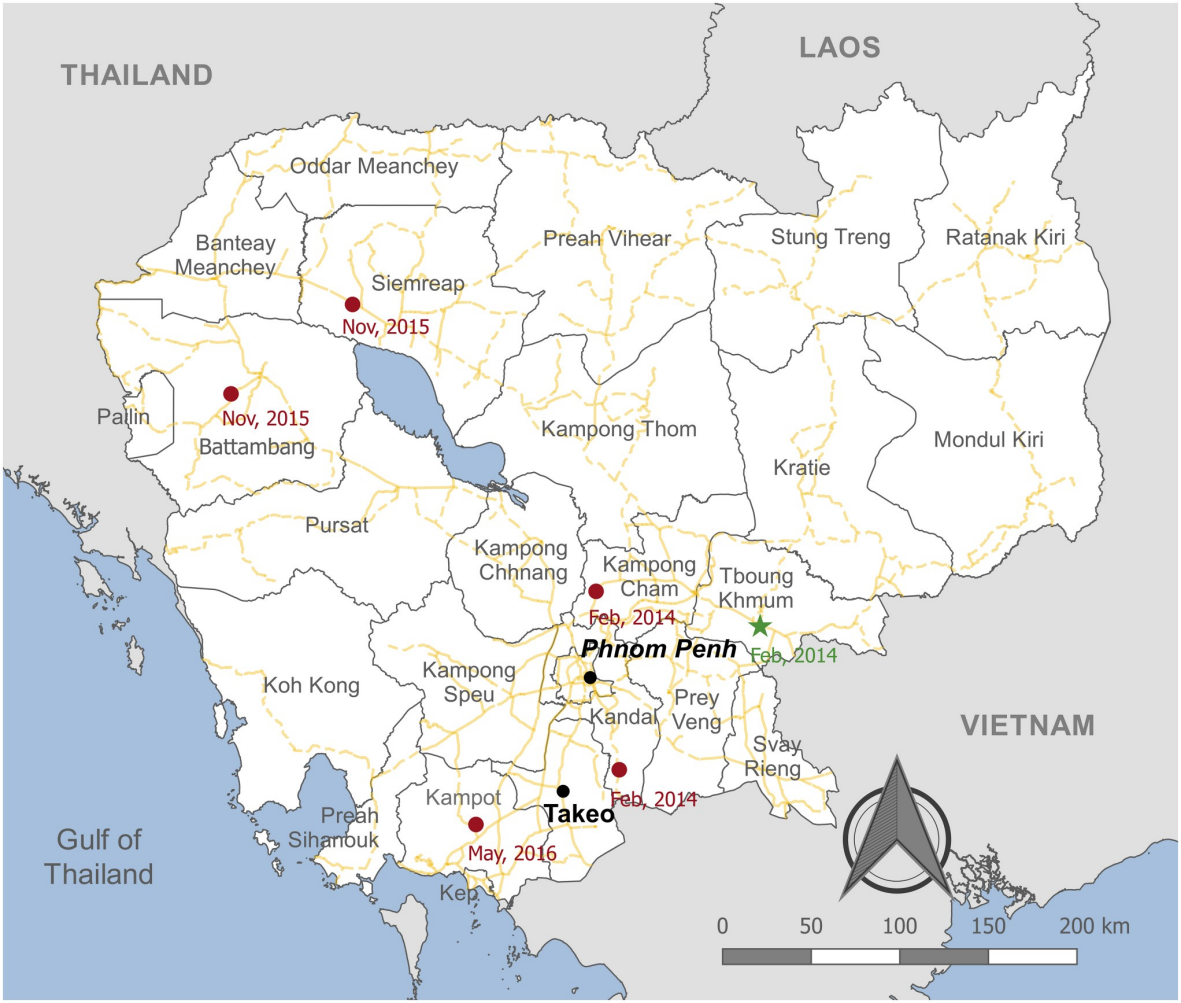
Map of Cambodia showing the locations of live bird market sampling sites, clade 2.3.2.1c AIV human cases and poultry outbreaks from 2014 to 2016. Poultry outbreaks were reported in the provinces: Battambang, Kampong Cham, Kampot, Kandal and Siem Reap. The approximate locations of each outbreak (n = 5) are shown by red circles, with the month and year of the outbreak listed below each site. The LBM surveillance sites were Orussey market in Phnom Penh and Takeo market in Takeo; both markets are indicated by black circles. The location of the single human AIV case caused by a clade 2.3.2.1c virus in Tboung Khmum is indicated by a green star. The map was produced using QGIS version 2.18.4 using public domain data obtained from Natural Earth (http://www.naturalearthdata.com/) [26].

Active surveillance was conducted at two prominent Cambodian LBMs: Phnom Penh, the capital city of Cambodia (Orussey market) and a provincial market in Takeo (Takeo market; Fig 1). Active surveillance was performed from 2015 to 2016, but not in 2014. The LBM surveillance strategies and AIV screening methods have been described previously for the 2015 study [25]. The methods used in the 2016 LBM study were the same as described for 2015, however the sampling strategy varied. In 2016, samples were collected solely from Orussey market and collections were performed around three Cambodian festival periods that are known to have high levels of AIV circulation [22]: Lunar New Year (February), Khmer New Year (April) and Pchum Ben (October).

Surveillance for human zoonotic influenza infections was conducted through: A) a country-wide influenza-like-illness sentinel surveillance system; B) severe acute respiratory illness (SARI) surveillance at two major paediatric hospitals (in Phnom Penh and Siem Reap); and C) event based surveillance focused on SARI cases with a history of contact with sick or dead poultry. All samples were screened as previously described [25] using influenza A virus and A(H5N1) qRT-PCR assays available at the International Reagent Resource (https://www.internationalreagentresource.org/Home.aspx).

### Viral isolation

Samples that had a high viral load (cycle threshold (CT) < 30 measured by qRT-PCR) were inoculated into embryonated chicken eggs (ECEs) for viral isolation. High viral load samples were prioritised as it becomes increasingly difficult to isolate viruses from samples with high CT values and the supply of ECEs in Cambodia is limited. Prior to inoculation, original samples were diluted 1:1 with antibiotics (a penicillin-streptomycin solution) and passed through a filter (0.22 μM) to prevent bacterial growth. The filtered solution was injected into the allantoic cavity of 10 to 12 day ECEs and incubated at 35°C for 48 to 72 hours in a humid chamber. After incubation, ECEs were chilled for a minimum of 4 hours to kill the embryo and constrict blood vessels. A hemagglutination assay (HA) was performed on the collected allantoic fluid using 0.5% chicken red blood cells. To confirm the presence of influenza A virus in HA positive samples a qRT-PCR was performed targeting the influenza A MP gene. Samples with a low or negative HA titre (0 or < 8 HA units) were re-passaged in ECEs. Viral isolation was considered unsuccessful if after three successive passages the allantoic fluid in ECEs had no HA titre and tested negative for influenza A via qRT-PCR.

### Sequencing of viral isolates

Influenza A(H5N1) ECE isolates were shipped to the WHO Collaborating Centre for Reference and Research on Influenza, Melbourne, Australia to obtain whole genome sequences using the Ion Torrent Personal Genome Machine^™^ (Thermo Fisher Scientific, Waltham, MA, USA). RNA was extracted from isolates using the NucleoMag^®^ VET kit (Macherey-Nagel, Bethlehem, USA) that purifies RNA using a magnetic-bead based system. A single RT-PCR reaction was performed to amplify all eight viral genomic segments [27]. The concentration of the PCR products was assessed using Agilent 2200 Tapestation. PCR products were then normalised and fragmented using the Ion Xpress^™^ Plus Fragment Library Kit (Thermo Fisher Scientific) to produce a 200 base-read library. The fragmented library was purified using the Agencourt^®^ AMPure^®^ XP Kit (Beckman Coulter, Brea, CA, USA) and barcoded using both the Ion Xpress^™^ P1 Adapter and a specific Ion Xpress^™^ Barcode (Thermo Fisher Scientific) for each individual sample. The barcoded library was pooled, purified and quantified by qPCR with the Ion Library TaqMan^™^ Quantitation Kit (Thermo Fisher Scientific). A 20 pM library was used for the emulsion PCR to obtain template positive, enriched Ion Sphere Particles^™^ (ISPs). The emulsion PCR was performed using the Ion PGM^™^ HiQ^™^ View OT2 Kit and run on the Ion OneTouch^™^ 2 Instrument (Thermo Fisher Scientific). The enriched ISPs were then loaded onto a 318^™^ Chip v2 (Life Technologies, Carlsbad, CA, USA) and run on the Ion Torrent PGM (Thermo Fisher Scientific).

Quality control of the resulting NGS reads was performed using CLC Genomic Workbench v10 (Qiagen, Valencia, CA, USA). Reads less than 50 nucleotides in length were removed and the remaining sequences were trimmed to only contain reads with a minimum Phred score of 20. High quality reads were then assembled to a reference genome. Any sequences with low coverage or gaps were completed using Sanger sequencing. A combination of segment specific primers and universal primers were used to amplify gene products for Sanger sequencing. PCR products were sequenced using Big Dye Terminator Reaction Mix (Thermo Fisher Scientific) on an ABI 3500xL Genetic Analyzer. Consensus sequences were generated and collated with available NGS data using Geneious^®^ 9.1.8 (Biomatters Ltd, Newark, NJ, USA). All sequencing accession numbers for the viral sequences generated and/or utilized in this study are listed in S1 Table.

### Phylogenetic analysis

AIV sequences used in the phylogenetic analysis were downloaded from GISAID or GenBank [28], curated and aligned using MAFFT v7.308 [29]. Each gene was tested for evidence of recombination using Genetic Algorithm for Recombination Detection (GARD) [30], using the datamonkey webserver [31]. Maximum Likelihood (ML) trees were estimated for each gene segment using IQ-Tree [32], under the General Time Reversible nucleotide substitution model with a gamma rate of heterogeneity (GTR+ Γ). Phylogenetic support was estimated using 1,000 ultrafast bootstrap replicates [33,34]. ML trees were visualized with the graphical viewer FigTree v1.4.3 [35].

A Bayesian phylogenetic analysis using the Markov chain Monte Carlo (MCMC) framework was performed for the HA and NA genes of Cambodian A(H5N1) clade 2.3.2.1c viruses using BEAST v1.8.4 [36] run using the CIPRES Science Gateway web portal [37]. Viral collection dates were parsed with variable precision. The analysis was run using an uncorrelated lognormal relaxed molecular clock [38] with the SRD06 nucleotide substitution model [39]. This model separates codons into two partitions, one containing the first and second codon position and the second partition containing the third codon position. The GTR + Γ substitution model was then applied to each partition [34]. Change in relative genetic diversity was estimated through time using a Bayesian skyride analysis utilising a Gaussian Markov Random Field (GMRF) smoothing prior [40]. For each gene, two independent analyses were performed for 100 million generations sampled to produce 10,000 states. A summary maximum clade credibility (MCC) tree was produced using TreeAnnotator v1.8.4 with 10% of the burnin removed. MCC trees with confidence intervals were visualised using FigTree v1.4.3 [35]. The GMRF plot was produced using Tracer v1.5.

HA gene lineages were defined by using nomenclature from the WHO/OIE/FAO A(H5N1) Evolution Working Group [21]. MP and internal gene lineages were described in a manner similar to previous reports [41–43]. Lineages were assigned based on gene ML phylogenies. Internal gene lineage designations consisted of the genomic segment number followed by a letter specific for each lineage e.g. gene: PB2, lineage A: 1A (S2a–S2g Figs). For genotypes identified, a two-letter country designation was provided (KH for Cambodia) followed by a systematic number. Genotyping was performed in this manner as it is similar to conventions used in Vietnam and allows us to easily compare A(H5) genetic constellations circulating between the two regions.

### Reassortment analysis

Phylogenetic congruence was used to investigate the occurrence of viral reassortment in Cambodian A(H5N1) viruses. ML phylogenetic trees were produced for all eight viral segments, as mentioned previously, and the topological position of each virus was tracked across the phylogenies. Incongruence is evident when there is deviation in the phylogenetic topology of individual viruses and can indicate putative reassortment events.

### Molecular analysis

The Centers for Disease Control and Prevention (CDC) have created a molecular inventory describing mutations and features in A(H5N1) and their overall effect on viral fitness [44]. This inventory was used in conjunction with an updated inventory of AIV molecular markers produced by Suttie et al., 2019 [45] to screen the Cambodian A(H5N1) clade 2.3.2.1c viruses for mutations of interest. Post translation modifications such as N-linked glycosylation sites were predicted for HA and NA using the NetNGlyc1.0 Server [46]. The numbering systems used throughout this text, unless otherwise specified, are: H5 for HA, N1 for NA. Internal genes are numbered relative to mature proteins from A/Vietnam/1203/2004(H5N1), however deletions in NA and internal gene segments are numbered relative to A/goose/Guangdong/1/96 [47].

### Selection pressure

To analyse the site specific selection pressures acting on each gene of the Cambodian A(H5N1) clade 2.3.2.1c viruses the ratio of non-synonomous substitutions (dN) and synonymous substitutions (dS), the dN/dS ratio denoted omega (ω), was computed using the HyPhy software package [48] accessed through the datamonkey webserver [31]. Selection is interpreted based on the value of ω, when ω < 1 sites are considered to be under negative selection, ω > 1 indicates positive selection and ω = 1 indicates neutrality. To perform this analysis, a combination of four methods: fixed-effects likelihood (FEL), fast unconstrained Bayesian approximation (FUBAR), mixed effects model of evolution (MEME) and single-likelihood ancestor (SLAC) were used [49–51]. FEL, FUBAR and SLAC estimate sites experiencing pervasive or diversifying selection pressure. Whereas, MEME detects pervasive or episodic positively selected codons. The analysis was based on the nucleotide alignment, the ML phylogeny and the best-fit nucleotide substitution model for each gene. To limit the number of false-positives, statistically significant sites (FEL, MEME and SLAC p-value <0.1 and FUBAR with a posterior probability ≥0.90) that were identified using more than one method were considered valid.

### Neuraminidase activity assay

A NA activity assay was performed to determine the appropriate dilution of each sample to use for the NAI susceptibility testing as described previously [52]. Briefly, gamma irradiated samples were serially diluted 2-fold and MUNANA substrate was added in a 1:1 volume ratio. After an incubation period of 1 hour at 37°C the reaction was stopped by adding a solution of 0.1 6M NaOH in absolute ethanol. Fluorescence was measured using a Fluoroskan Ascent^™^ Microplate Fluorometer (Thermo Scientific) with standard filters for excitation (λ 360 nm) and emission (λ 448nm). The NA enzymatic activity was plotted and a viral dilution in the linear region of the curve was used to perform the NAI inhibition assay.

### Neuraminidase inhibition assay

The susceptibility of each virus to four NAI drugs (oseltamivir, zanamivir, laninamivir and peramivir) was quantified by comparing the fluorescence of the uninhibited virus compared to the virus after it had been incubated with varying concentrations of NAI drugs (final drug concentrations ranged from 0.01 nM to 10,000 nM). Briefly, samples were diluted according to NA activity assay results and incubated at room temperature for 45 minutes with the varying drug concentrations. NA activity was then measured as above. The drug concentration that inhibited NA enzymatic activity by 50% was defined as the IC_50_ value, and this was calculated using the JASPR^™^ v1.2 software (CDC, Atlanta, GA, USA).

The WHO Influenza Antiviral Working Group have established criteria for determining AIV susceptibility to NAI drugs based on the fold change in IC_50_ values [53]. Viruses are described as having either: normal (< 10-fold change), reduced (10 to 100-fold increase) or highly reduced (> 100-fold increase) inhibition. Three control viruses (A/Perth/82/2015, A/Osaka/180/2009 and B/Memphis/20/96-R152K) that have established IC_50_ ranges were included in the NAI assay in duplicate. If the mean IC_50_ value of each control virus was within the accepted range, the assay was considered valid.

## Results

### Viral isolation

During 2014–2016, 42 A(H5N1) clade 2.3.2.1c viruses were isolated from poultry (21 chickens and 21 ducks) in Cambodian LBMs (S1 Table). Of these, 38 were obtained from an LBM surveillance study conducted in 2015 [54] and four from spot-testing in Cambodian LBMs during 2016. Additionally, partial A(H5N1) genomes from nine environmental samples, collected from containers used to wash poultry carcasses in 2015 were also included in the analysis [25]. In addition, 16 clade 2.3.2.1c viruses were isolated from five poultry outbreaks during passive surveillance of poultry illnesses and deaths. In total, 67 clade 2.3.2.1c viruses from Cambodian LBMs and poultry outbreaks were analysed in this study (S1 Table), with 42 full genome sequences and 25 partial sequences.

Since the introduction of A(H5N1) clade 2.3.2.1c into Cambodia only one human case has been detected (February 2014) [18]. This virus (A/Cambodia/Y0219302/2014) could not be isolated due to low viral load in the clinical samples. As such, only limited HA and full length NP genes could be generated for this virus (S1 Table). The HA and NP sequences of this human sample were also included in this study.

### Sequence analysis of A(H5N1) clade 2.3.2.1c genes

**Fig 2:**
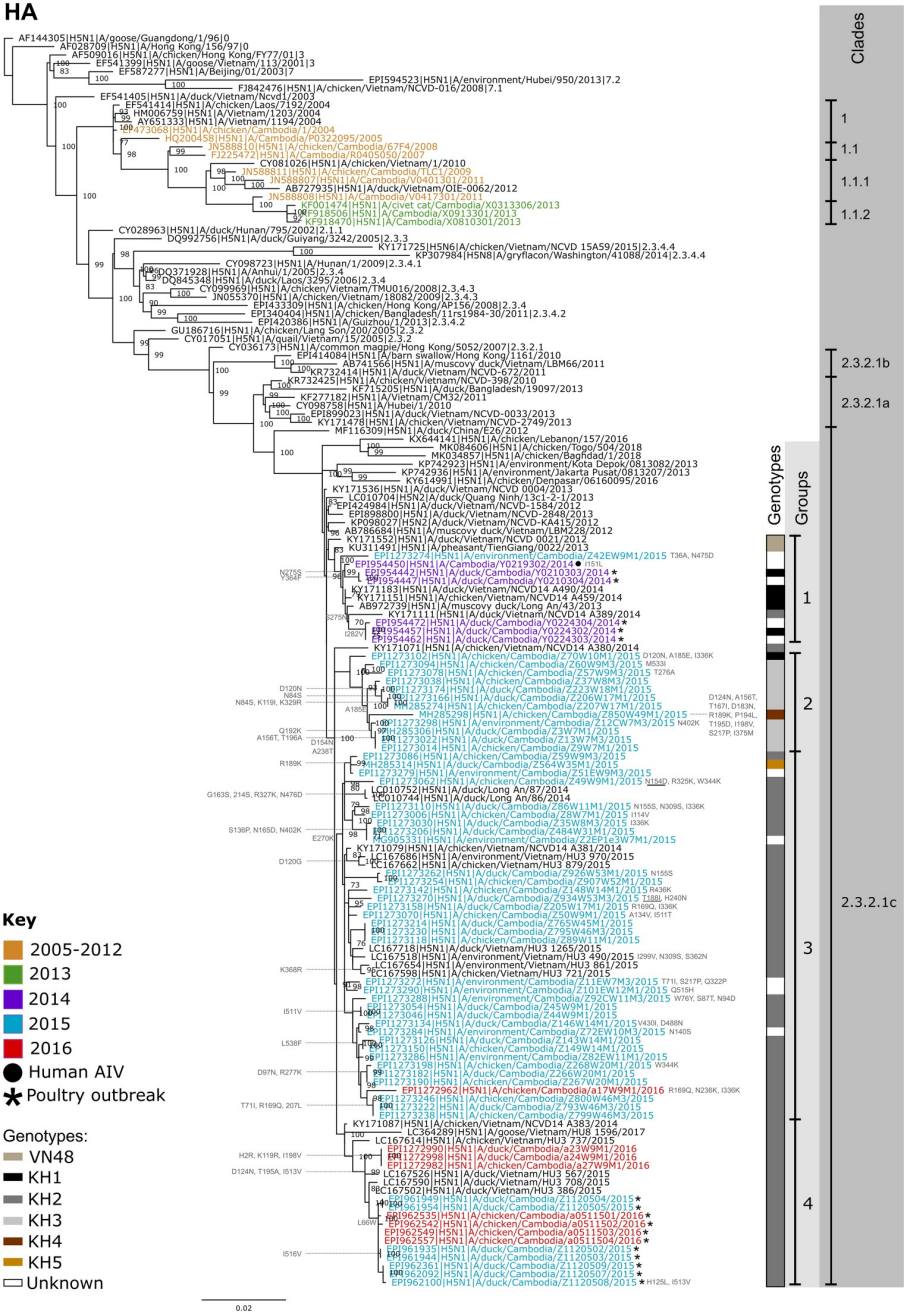
Maximum likelihood phylogenetic tree for the HA gene of A(H5) AIVs. The tree was generated with IQ-Tree using GTR+ Γ and 1,000 ultrafast bootstrap replicates. Cambodian viruses are coloured based on the year they were collected. Viruses detected prior to 2013 are coloured orange, viruses from 2013 are green, viruses from 2014 are purple, 2015 are blue and 2016 are red. Viruses detected during poultry outbreaks are indicated by an asterisk (*) and the single clade 2.3.2.1c Cambodian human A(H5N1) is indicated by a black circle next to the taxa name. Viral clades, HA groups and genotypes are indicated on the right hand side of the tree. Viral clades not listed on the right hand side of the tree have been appended to the end of taxa names. Amino acid differences relative to the closest related WHO candidate vaccine virus (A/duck/Vietnam/NCVD-1584/2012) are shown next to the phylogeny in grey. Mutations listed at branches on the left side of the tree prevail in descendant viruses. Mutations listed next to viral taxa on the right side of the tree are found in the individual virus. Underlined mutations are those that have been previously reported to affect viral fitness. Bootstrap values of 70 or greater are displayed on nodes.

Sequence and phylogenetic analyses revealed that the Cambodian clade 2.3.2.1c HA genes formed a monophyletic clade with viruses identified in Vietnam from 2012 to 2017. The HA nucleotide sequence identity of all Cambodian 2.3.2.1c viruses from 2014 to 2016 ranged from 96.2 to 100% and were broadly separated into four main groups (Fig 2). HA group 1 was an outlier to the three other groups and contained all clade 2.3.2.1c viruses identified in 2014 (n = 6; including the human case) and a single virus from 2015. HA groups 2 and 3 contained the majority of isolates detected in the 2015 LBM study (n = 12 and 34, respectively). Additionally, a single isolate from the 2016 LBM study clustered with HA group 3. HA group 4 contained all of the A(H5N1) viruses from 2015 and 2016 poultry outbreaks (n = 7 and 4 respectively), as well as the majority (3/4) of the 2016 LBM viruses. The HA groups are closely related; Group 2 is the parental clade to groups 3 and 4, which are sister clades.

Bayesian estimates of the time to most recent common ancestor (TMRCA) for the HA gene of the Cambodian clade 2.3.2.1c viruses indicated that the six groups diverged from a common ancestor around April, 2012 with a 95% Highest Posterior Density (HPD) interval ranging from February to June, 2012 (S1 Fig). The viruses from HA groups 3 and 4, along with viruses sampled in Vietnam during 2014–2017, shared a more recent common ancestor around April 2013 (95% HPD: November 2012 to August 2013).

Similar to the HA gene, the NA and internal genomic segments all clustered with Vietnamese viruses identified from 2012 to 2017. The defined HA groups were also relatively well conserved for NA and the internal genomic segments (S2a–S2g Figs). The nucleotide sequence identity of the Cambodian A(H5N1) genes were as follows; NA: 96.9–100%, PB2: 96.6–100%, PB1: 88.7–100%, PA: 95.8–100%, NP: 94.1–100%, MP: 85.6–100%, NS: 93.2–100%. The lower sequence identity of the PB1 and MP segments were the result of reassortment between A(H5N1) and A(H9N2). Excluding the A(H9N2) reassortant viral segments the sequence identity of PB1 and MP ranged from 97–100% and 95.4–100% respectively.

The human clade 2.3.2.1c virus (A/Cambodia/Y0219302/2014) HA gene was 99.5% identical to that of two viruses detected in poultry around the same period (A/duck/Cambodia/Y0210303/2014 and A/duck/Cambodia/Y0210304/2014). These three viruses differed by only eight bases resulting in two amino acid changes in the human AIV, I151L and F364Y. Neither mutation has been documented to be associated with a significant increase in viral fitness. The NP gene for A/Cambodia/Y0219302/2014 was 99.7% identical to A/duck/Cambodia/Y0224302/2014 and differed by only five synonymous nucleotide changes.

### Detection of reassortment in A(H5N1) clade 2.3.2.1c viruses

**Fig 3:**
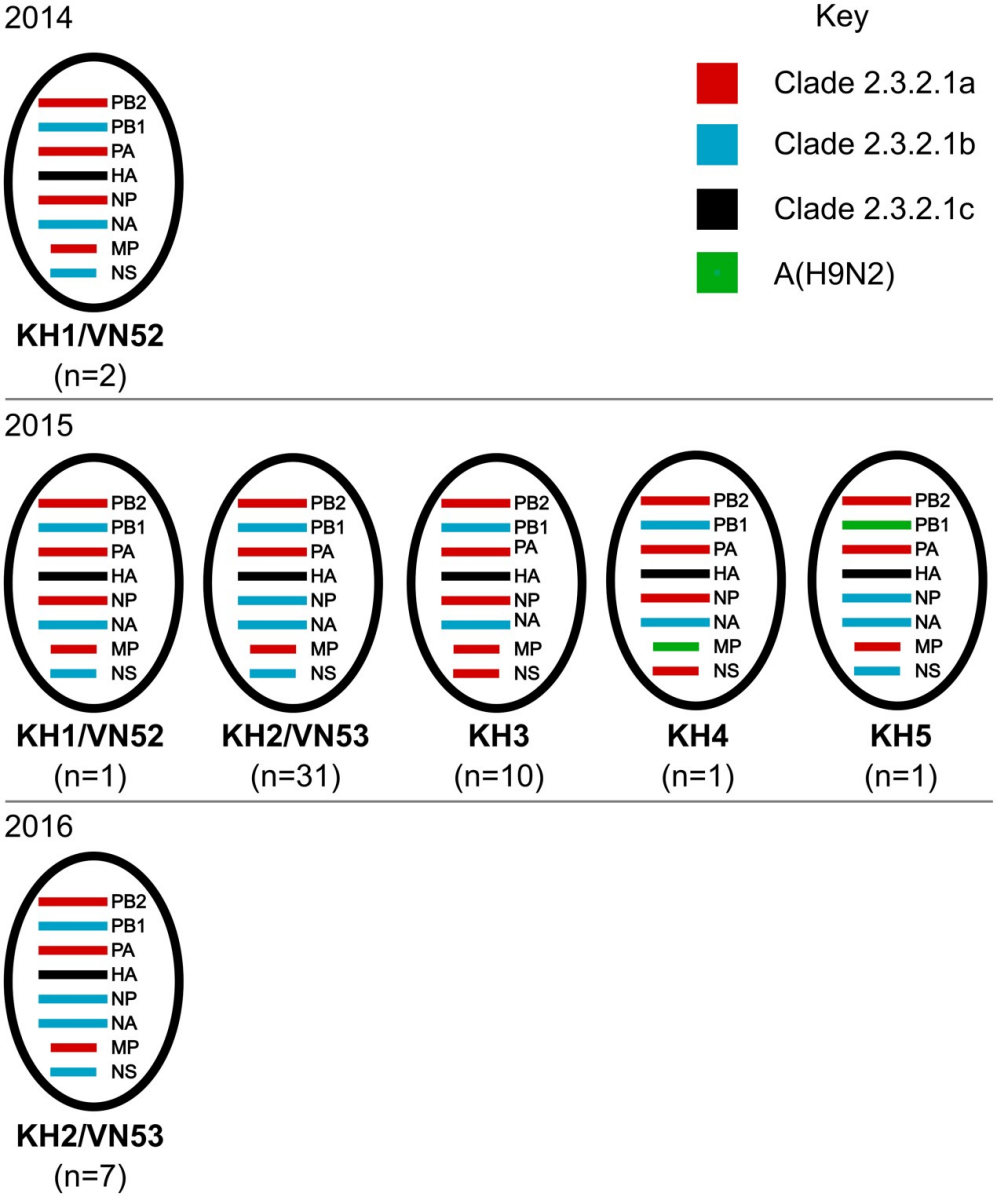
Genotypes of highly pathogenic A(H5N1) viruses detected in circulation in Cambodia from 2014 to 2016. Genomic segments are colour coded to show their possible ancestry. A(H5) clade 2.3.2.1a is shown in red, clade 2.3.2.1b in blue and clade 2.3.2.1c in black. Segments from A(H9N2) viruses are shown in green. Designated KH genotypes (KH1-KH6), any Vietnamese genotype equivalents (VN52, VN53) and the number of Cambodian isolates detected are shown below the associated genome constellations.

**Fig 4:**
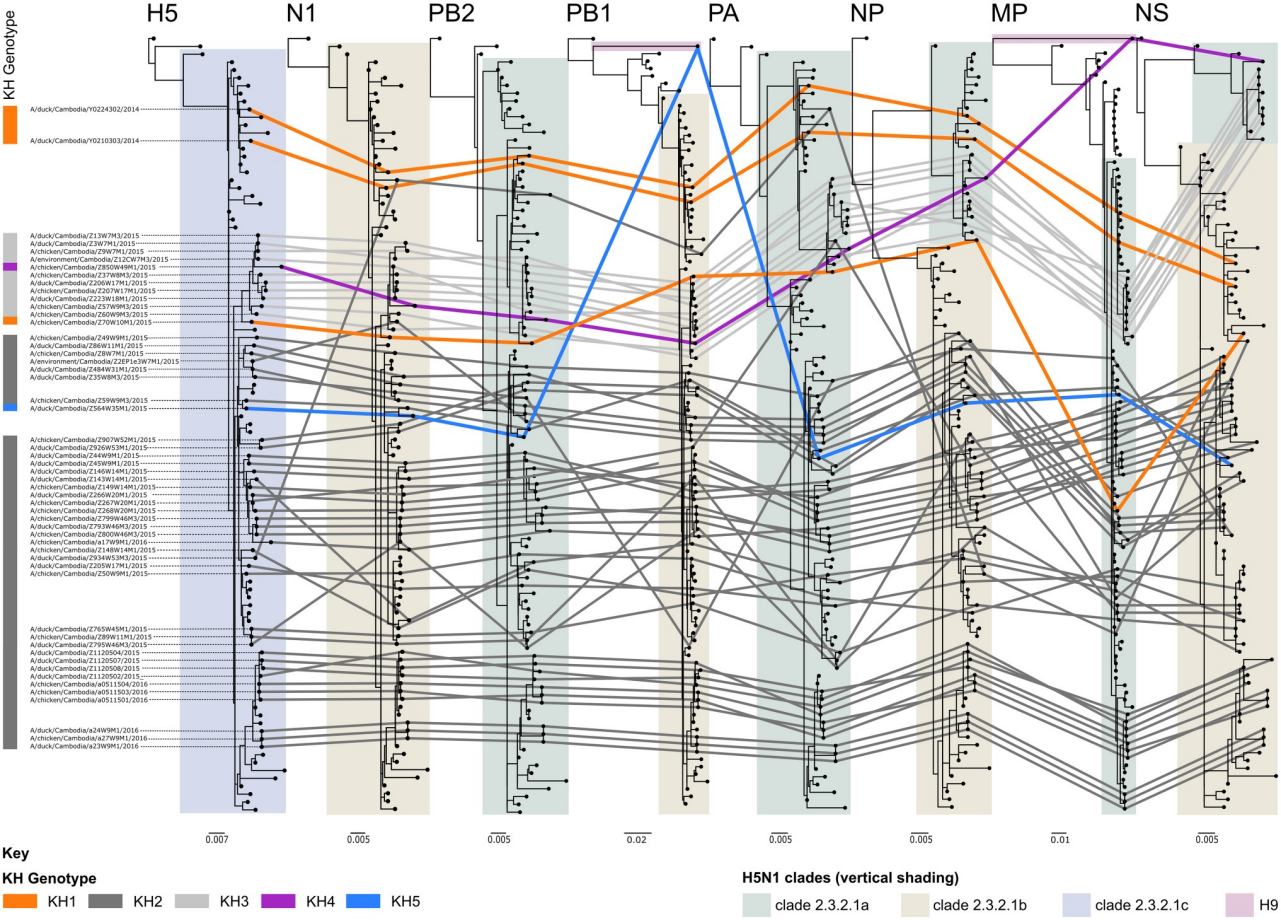
Phylogenetic congruence map of Cambodian A(H5N1) viruses. ML phylogenetic trees were produced for each genomic segment. The scale bars indicate the number of nucleotide substitution per site. For each Cambodian A(H5N1) virus, links have been drawn to connect phylogenetic position of individual viruses across all eight genomic segments. For visual clarity the links are colour coded based on viral genotypes. Viral designations and genotypes are indicated on the left side of the figure. The KH1 genotype is coloured orange, KH2 is dark grey, KH3 is light grey, KH4 is purple and KH5 in blue. Additionally, the putative ancestry of the HA, NA, MP and internal gene segments are shown by vertical shading of each ML tree. Clade 2.3.2.1a is shaded in green, 2.3.2.1b in brown, 2.3.2.1c in blue and A(H9N2) in mauve.

The phylogenetic analyses performed for all eight genomic segments showed no evidence of recombination, however there was evidence of numerous reassortment events. Analysis of the genomic constellations shows five genotypes (KH1-KH5) of clade 2.3.2.1c viruses circulated in Cambodia from 2014 to 2016 (Fig 3). Genotypes KH1 and KH2 were originally identified in Vietnamese poultry and originated from reassortment events between clades 2.3.2.1a (A/Hubei/1/2010-like), 2.3.2.1b (A/barn swallow/Hong Kong/1161/2010-like) and 2.3.2.1c viruses [41]. KH1 was the only clade 2.3.2.1c genotype detected in 2014 (n = 2), with a single isolate detected in 2015 and was consistent with the Vietnamese genotype VN52. ML trees showed that all KH1 PB2, PA and NP segments clustered with clade 1.1.2 reassortant viruses detected in Vietnam and Cambodia in 2013 that obtained their NA and internal genomic segments from clade 2.3.2.1a viruses (S2b, S2d and S2e Figs). The KH1 MP genes were also from the A/Hubei/1/2010-like lineage, though only the KH1 viruses detected in 2014 cluster with clade 1.1.2 reassortant viruses (S2f Fig). The phylogenetic congruency map shows the KH1 virus from 2015 acquired its MP gene through a separate reassortment event (Fig 4). The NA, PB1 and NS segments were from the A/barn swallow/Hong Kong/1161/2010-like lineage. The KH1 genotype has not been detected since March, 2015.

KH2 was the most frequently identified genotype in 2015 (n = 31) and the sole genotype detected in 2016 (n = 7; Fig 3). The genomic constellation of KH2 –strictly identical to the Vietnamese genotype VN52 –differed from KH1 only in its NP gene, which was from the A/barn swallow/Hong Kong/1161/2010-like lineage. A single KH2 virus, designated A/duck/Cambodia/Z934W53M3/2015, had an atypical PB2 gene (relative to this genotype) that clustered with clade 1.1.2 reassortant viruses (S2b Fig). The phylogenetic congruency map showed that the KH2 genotype also gave rise to KH5 (n = 1), that acquired a PB1 segment from circulating A(H9N2) AIVs (Fig 4).

The second most prevalent genotype in 2015 was KH3 (n = 10), produced from the reassortment of clade 2.3.2.1c and clade 1.1.2 reassortant viruses. The NA and PB1 segments cluster with clade 2.3.2.1c viruses (A/barn swallow/Hong Kong/1161/2010-like). Whereas the PB2, PA, MP and NS genes all stem from clade 1.1.2 reassortant viruses (S2b, S2d, S2f and S2g Figs). The NP genes of KH3 viruses also stem from clade 2.3.2.1a viruses, forming sister clades with the 1.1.2 reassortant viruses (S2e Fig, group 5A). The KH3 genotype was last detected in March, 2015 though it may have been circulating undetected for a longer period as this genotype gave rise to KH4 (n = 1) identified in November 2015 (Fig 3). The KH4 genotype differed from KH3 only in the clustering of the MP gene as it was acquired from A(H9N2).

### Spatial phylogenetic patterns

The Cambodian A(H5N1) clade 2.3.2.1c AIVs detected within the LBM system do not show strong spatial segregation. The two main LBM sampling sites in Cambodia were Orussey market and Takeo market, 76 km apart by road (Fig 1). HA groups 2 and 3 that collectively account for 92% (n = 47/51) of the LBM AIVs analysed in this study, all contain viruses from both LBMs with no strong subgrouping based on sampling location. Comparatively, HA groups 1 and 4 contain viruses identified from Orussey market or from poultry outbreaks in multiple Cambodian provinces. HA group 1 contains viruses from two poultry outbreaks in Kampong Cham and Kandal from February 2014. These viruses are closely related, belonging to the same cluster, however they form clearly distinguishable subgroups based on outbreak location. Comparatively, HA group 4 contains viruses from three different poultry outbreaks from 2015 and 2016 with less distinct spatial clusters. This is interesting, considering in some instances these viruses were detected approximately six months and over 400 km apart.

### Phylodynamics

**Fig 5:**
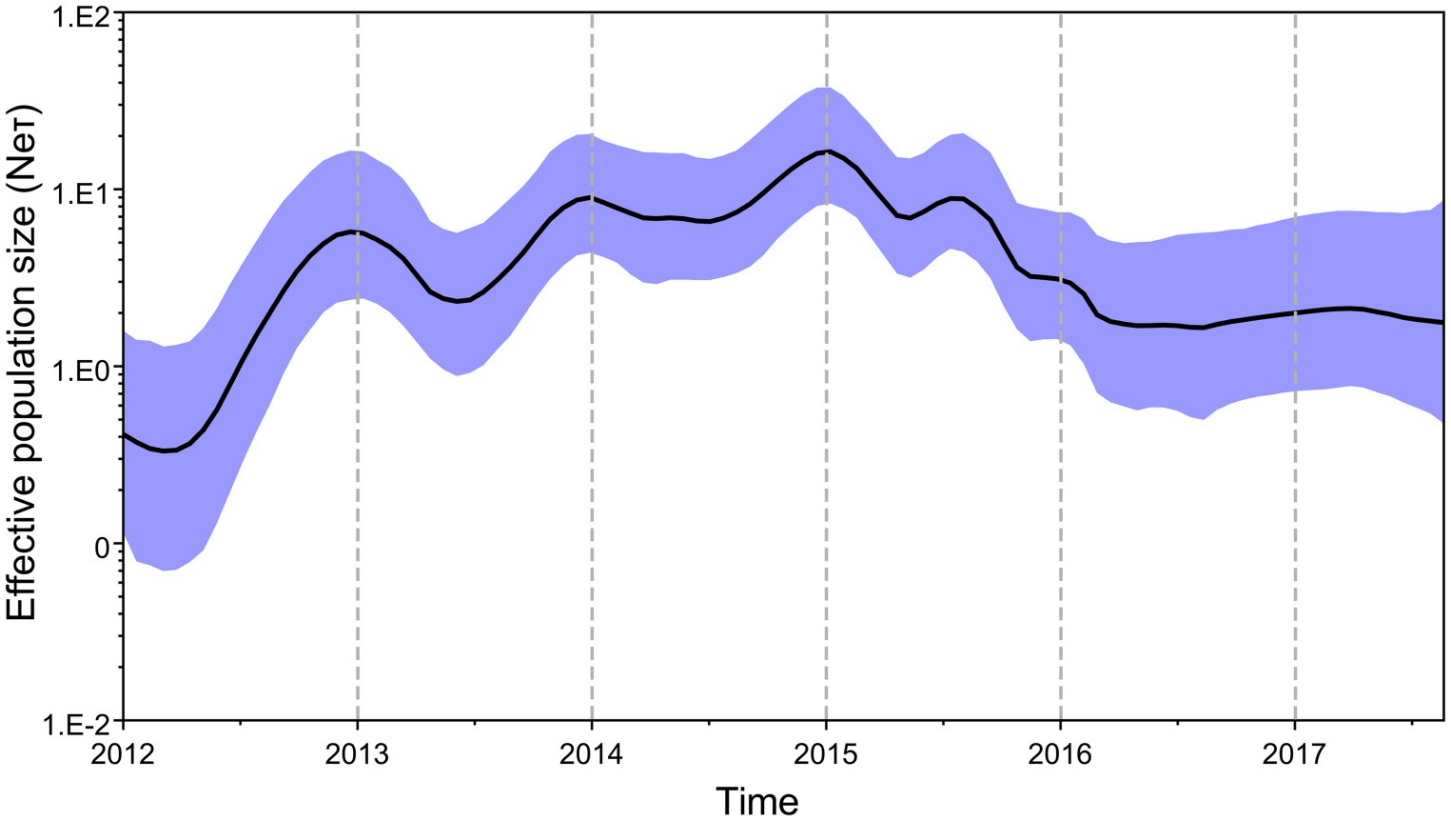
Bayesian skyride analysis of Cambodian clade 2.3.2.1c AIV genetic diversity. Genetic diversity was estimated using the Gaussian Markov Random Field (GMRF) model using the HA BEAST dataset. The x-axis measures time in years and the y-axis is an estimate of genetic diversity calculated from Neτ (effective population size and the generation length in years) shown in log scale. The median estimate of genetic diversity over time is shown as a solid black line and the purple shading represents the 95% HPD intervals.

The Bayesian skyride analysis of Cambodian, and closely related Vietnamese, clade 2.3.2.1c A(H5N1) viruses shows fluctuations in genetic diversity from 2012 to 2017. From 2012 to 2013, when clade 2.3.2.1c A(H5N1) was initially detected in Vietnam there was a marked increase in relative genetic diversity (Fig 5) [41]. This is typical when pathogens are newly introduced into a geographical region with a naïve population. From 2013 to 2016, genetic diversity oscillated with peaks occurring each year around December and January. These months have been associated with higher prevalence rates of AIVs in Cambodian LBMs [22,54]. From 2012 to 2015, the effective population size was generally increasing. In 2015, there were more fluctuations in genetic diversity and throughout the year the effective population size was gradually declining. In 2016, after the initial peak around January, genetic diversity became relatively stable, with a slight increase in the effective population size in the latter half of the year.

### Cambodian A(H5N1) clade 2.3.2.1c viruses display molecular markers of pathogenicity, adaptation and transmission

Markers of increased virulence in mammalian and/or avian models of disease were identified in the PB2, PB1-F2, HA, NP, NA, M1, M2 and NS genes (summarised in Table 1, a full list of markers investigated is available in S2a–S2h Table). All Cambodian A(H5N1) clade 2.3.2.1c viruses analysed in this study (n = 68) possessed HA genes with multibasic cleavage sites characteristic of A(H5) HPAI viruses. HA sequences also contained mutations associated with an increase in sialic acid receptor binding to α2,6 human-type receptors, including: D94N, S133A, S155N, T156A, T188 and K189R. Though, numerous substitutions were also identified that are associated with a binding preference for α2,3 receptors (S2d Table). The well-known Q222L and G224S substitutions associated with increased binding to α2,6 receptors and decreased α2,3 binding were not observed [55,56].

**Table 1:** Summary of amino acid substitutions in Cambodian A(H5N1) viruses associated with changes in viral fitness.

Protein	Phenotype	Mutation/Motif	Cambodian Isolates (%)	References
PB2	Increased virulence in mice	I63T	I (100)	[57]
	Increased polymerase activity, replicative capacity, virulence in mice and contact transmission in guinea pigs	E627K	E (99), Q (1)	[11]
		D701N	D (100)	[58]
PB1	Increased polymerase activity and virulence in mice	D622G	G (100)	[59]
PB1-F2	Truncations to the 90 aa protein increase AIV pathogenicity in chickens	Truncation	25 aa (4)	[60,61]
			57 aa (94)	
			90 aa (2)	
PA-X	Truncations to the 253 aa protein increases A(H5) viral replication and virulence in mice, chickens and ducks	Truncation	253 aa (100)	[62–64]
HA	Multibasic cleavage site can increase viral pathogenicity	Multibasic	PPRERRRKR/GLF (1)	[65]
			PQREKRRKR/GLF (1)	
			PQREKRRKR/GLF (1)	
			PQRERRRKR/GLF (94)	
			PQRERRRRR/GLF (3)	
	Increased in specificity for α2,6 human-type receptors	D94N	N (99)	[66]
		S133A	A (100)	[67]
		S155N	N (97)	[68]
		T188I	I (1)	[67]
		K189R	R (94)	[68]
		Q222L	Q (100)	[55,56]
		G224S	G (100)	
	Increased in specificity for α2,6 human-type receptors, increased transmission in guinea pigs	T156A	A (94)	[68]
NP	Increased replication in avian cells and virulence in chickens	M105V	V (100)	[69]
		A184K	K (100)	[70]
NA	Enhanced virulence in mice	49–68 deletion	49–68 deletion (100)	[71]
M1	Enhanced virulence in mice	N30D	D (100)	[72]
		T215A	A (100)	
	Enhanced virulence in mice, chickens and ducks	I43M	M (100)	[73]
M2	Increased resistance to amantadine and rimantadine	A30T	T (2)	[74,75]
		S31N	N (2)	
NS1	Decreased antiviral response and increased virulence in mice	80–84 aa deletion	80–84 deletion (100)	[76]
		P42S	S (100)	[77]
		D87E	E (77)	[76]
		L98F	F (100)	[78]

A number of the mutations identified in the NA, MP and NS genes are conserved in contemporary A(H5N1) viruses [44]. For instance, the NA and NS1 stalk deletions, and the NS1 substitutions: P42S, D87E, L98F and I101M residues that have been shown to increase AIV virulence in mice [76–80]. In the MP gene, all Cambodian A(H5N1) viruses contained the M1 N30D and T215A residues associated with increased mammalian pathogenicity *in vivo* [72]. No major molecular markers associated with AIV adaptation to mammalian species were identified in the polymerase proteins, such as PB2 E627K or D701N.

### Analysis of selection pressures

The site-specific selection pressures acting on each gene of the Cambodian clade 2.3.2.1c viruses were analysed using a combination of four methods (FEL, FUBAR, MEME and SLAC) that calculate omega (ω). The majority of codons in all genes of the Cambodian A(H5N1) clade 2.3.2.1c viruses were under either neutral or purifying selection pressure (S3 Table). A total of six codons were detected to be under positive selection, including: HA (169), NA (74), PB2 (107, 339) and PB1 (54, 739; S3 Table). The HA and NA codons do not lie in major antigenic sites. Whereas, the PB1 codons 54 and 739 are part of PB1-PA or PB1-PB2 binding domains, respectively [81,82]. Substitutions at all the detected codons, excepting PB2 339, have not been shown to affect viral fitness. The positively selected codon in PB2 at position 339 is located on the surface of A(H5N1) PB2 at the edge of a putative cap binding site [83]. At this site Cambodian clade 2.3.2.1c viruses predominantly contained threonine (339T), with a single isolate from 2015 containing methionine at this site (339M). Reports on the effect of PB2 mutations at position 339 on viral virulence vary and so the effect of mutations at this codon on viral fitness remain unclear [84–86].

### Post translational modifications—N glycosylation

A total of nine N-glycosylation sites were predicted from the HA sequences of the Cambodian clade 2.3.2.1c A(H5N1) viruses (S4 Table). Of these, five glycosylation sites predicted in the HA2 stem domain were identified in all Cambodian viruses, including: ^11^NNS, ^23^NVT, ^286^NSS, ^484^NGT and ^543^NGS [87]. In the HA1 subunit four additional glycosylation sites were identified: ^140^NSS, ^165^NNT, ^236^NDT and ^273^NCS. The HA N-glycosylation sites have all been identified in A(H5N1) viruses previously [87]. The glycosylation sites in the HA2 stem domain and at position 165 are highly conserved. The ^140^NSS glycosylation site emerged in clade 2.3.2.1 viruses and occurs in a HA antigenic site at epitope B [87]. From the Cambodian NA sequences five glycosylation sites were predicted, including: ^28^NIT, ^35^NHS, ^68^NSS, ^126^NGT and ^215^NGS (S4 Table). The glycosylation sites ^126^NGT and ^215^NGS were conserved in all Cambodian A(H5N1) NA proteins. The ^68^NSS glycosylation site was predicted for all, barring one, NA segments. These three sites are conserved in A(H5N1) viruses [87]. Whereas, the remaining two glycosylation sites predicted in the Cambodian viruses are less common: ^35^NHS (n = 13), ^28^NIT (n = 3) (S4 Table) [87].

### Molecular prediction and susceptibility to antiviral drugs

Two Cambodian A(H5N1) viruses belonging to clade 2.3.2.1c had predicted resistance to adamantanes. The first virus, designated A/chicken/Cambodia/Z50W9M1/2015, had an M2 protein A30T substitution. The second virus, designated A/chicken/Cambodia/Z850W49M1/2015, was a reassortant with all internal genes from A(H5N1) viruses and an MP from circulating A(H9N2) viruses, and has been described previously [88]. The A(H9N2) acquired M2 protein contained the S31N substitution known to confer resistance to adamantanes. The A30T and S31N substitutions are well known and many experimental studies have shown these single amino acid substitutions render AIVs highly resistant to adamantanes.

The Cambodian clade 2.3.2.1c viruses did not contain any known markers indicative of resistance to neuraminidase inhibitors (NAI; S2f Table). However, there were two variants detected at position 129: V129I (n = 3) and V129D (n = 1). The mutation V129A was previously reported to mildly decrease AIV susceptibility to zanamivir [89]. The effect of V129I/D substitutions on AIV susceptibility to NAI drugs is unknown. To confirm NAI sensitivity, a subset of thirty isolates were tested against four NAIs (oseltamivir, zanamivir, peramivir and laninamivir) with no resistance detected (S5 Table). All three viruses with V129I substitution were included in the phenotypic testing and no decrease in susceptibility to NAI drugs was observed. The effect of V129D could not be investigated as no isolate was available for this sample. The mean fold change in IC_50_ (nM) values for oseltamivir, peramivir and zanamivir were 1.0 and for laninamivir 1.18 (S5 Table).

## Discussion

AIVs present a threat to agriculture and could potentially cause the next pandemic, therefore it is vital to continually monitor their circulation and evolution. A(H5N1) HPAI viruses were introduced into Cambodia in 2004. They have since become endemic in domestic poultry, causing substantial economic hardship. Continual circulation of A(H5N1) HPAI viruses in Cambodian poultry is also concerning as humans that live and work in close contact to poultry are at risk of zoonotic transmission due to the lack of biosecurity. Therefore, we sought to investigate the phylogenetic and molecular traits of Cambodian A(H5N1) viruses collected from LBM studies, poultry outbreaks and human infections from 2014 to 2016.

Between 2014 to 2016, five A(H5N1) clade 2.3.2.1c poultry outbreaks were reported in Cambodia (Fig 1). In reality the number of AIV outbreaks is likely to be much higher due to reluctance in reporting [90]. Active surveillance was also performed at Cambodian LBMs in 2015 and 2016. Previous studies have demonstrated that the Cambodian LBMs have a high prevalence of AIVs with a diverse range of AIV subtypes detected [22,54,91].

Since March 2014, A(H5N1) viruses detected in Cambodia have exclusively been of clade 2.3.2.1c [25]. Clade 2.3.2.1c viruses are one of three viral subclades (2.3.2.1a-c) that evolved from clade 2.3.2.1 and were initially reported in domestic poultry from Vietnam in 2012 [41,92]. However, reports show that clade 2.3.2.1c began to circulate widely throughout Asia from 2009 onwards [41,92]. Clade 2.3.2.1c viruses have been detected in both poultry and wild waterfowl species. The widespread circulation of clade 2.3.2.1c viruses in wild birds has facilitated their geographical dispersal, with clade 2.3.2.1c viruses reported in the Middle East, Europe and Africa [93–96]. Aside from avian species, this clade has also been detected in mammals, including big cats (a lion and tiger from China) [97,98] and humans (China: A/Hong Kong/6841/2010; Cambodia: A/Cambodia/Y0219302/2014).

In the present study, we investigated the genetic diversity and evolution of Cambodian clade 2.3.2.1c viruses circulating between 2014 and 2016. The close phylogenetic and molecular association of 2.3.2.1c viruses detected in Cambodia and Vietnam suggests that these viruses circulate endemically between the two countries. Similar trends were observed for the introduction and evolution of clade 1 A(H5N1) HPAI viruses that were first detected in Cambodia in 2004 [99]. The close relationship between the Cambodian and Vietnamese viruses is unsurprising considering cross-border trade and farming of poultry between the two countries is known to occur [100]. It is also possible that the transmission of AIVs between Cambodian and Vietnam is facilitated by the movement of wild birds. However, the role of wild birds in the introduction of AIVs into Cambodia has not been investigated to date.

The Cambodian clade 2.3.2.1c viruses circulating between 2014 and 2016 were diverse, with five genotypes present over a 3-year period (KH1-KH5). Of these, the KH1 and KH2 genotypes were equivalent to VN52 and VN53, which circulated in Vietnamese poultry in 2013–2015 [42]. Whereas, KH3-KH5 were novel genotypes identified in Cambodia in 2015 that were produced from reassortment between A(H5N1) clades or with A(H9N2) viruses locally. The generation of novel AIV genotypes can have important public health implications. For instance, the acquisition of specific A(H9N2) internal genomic cassettes has been associated with an increase in zoonotic potential of A(H7N9) and A(H10N8) AIVs [101,102]. Reassortment between A(H5) viruses can also produce viruses of concern for human health. Indeed, this was demonstrated in Cambodia in 2013 with the emergence of the novel clade 1.1.2 reassortant viruses that caused numerous human A(H5N1) cases.

The phylogenetic analysis shows limited signs of spatial segregation. Viruses identified at LBMs from 2015 to 2016 cluster closely together in HA groups 2 and 3 (Fig 2). This is unsurprising considering the high degree of poultry movement within the LBM network and that poultry bought at Takeo market are commonly sold to individuals that transport the poultry to the capital city to resell at Orussey market (Fig 1). Spatial segregation was, at times, more evident in viruses detected during poultry outbreaks as seen from the distinct grouping of outbreak samples from Kampong Cham and Kandal, isolated in February, 2014. Comparatively, in outbreak samples detected in 2015 and 2016 spatial segregation was less evident despite the fact that these samples were collected approximately six months apart at sites that were, in some cases, separated by more than 400 km. These findings demonstrate the persistent circulation and spread of highly pathogenic A(H5N1) viruses in Cambodia outside of the LBM network.

As expected, all of the Cambodian A(H5N1) clade 2.3.2.1c viruses had multibasic HA cleavage site motifs characteristic of HPAI viruses. The Cambodian viruses contained six amino acid substitutions that have been shown to increase receptor binding preference for α2,6 human receptors, including: D94N, S133A, S155N, T156A, T188I and K189R [66–68,89]. Typically, five out of six of these mutations were detected. None of the viruses analysed contained the Q222L or G224S substitutions that are associated with mammalian adaptation. Most of the HA molecular sites associated with host specificity that were investigated are indicative of a preference for avian α2,3 receptors (S2d Table). The identified mutations associated with increased specificity for α2,6 are unlikely to switch the receptor specificity of the Cambodian A(H5N1) viruses entirely. No other mutations associated with AIV adaptation to mammals, such as E627K and D701N in PB2 [103,104], were identified in the internal genes of the Cambodian A(H5N1) viruses.

Post translational modifications, such as the N-glycosylation, are important for protein folding, maturation and biological functionality. In AIVs, the presence of N-glycans on the HA and NA can affect viral pathogenicity and virulence as N-glycans can shield antigenic sites enabling viruses to evade detection by the host immune system [105–107]. They have also been shown to affect HA receptor binding preference and cleavability [108,109]. The T156A mutation, detected in 64 out of 68 of the Cambodian clade 2.3.2.1c AIVs, removes an important glycosylation site in HA. The absence of glycosylation at position 154–156 is common in viruses that stem from clade 2.3.2.1 [87] and has been shown to increase viral affinity for α2,6 sialic acid receptors [68,110]. Furthermore, multiple independent studies have shown that mutations at these positions, such as N154D and T156A, are important factors in the transmissibility and pandemic potential of AIVs when combined with Q222L and G224S [11,12].

A small number of codons were determined to be under positive selection pressure, including: HA (169), NA (74), PB2 (107, 339) and PB1 (54, 739) (S3 Table). The NA codon 74 is part of the stalk region, PB1 codon 54 is part of the PB1-PA binding domain, 739 is part of the PB1-PB2 binding domain [82] and PB2 codon 339 is located at the edge of a PB2 cap binding site [83]. Substitutions at all the detected codons, excepting PB2 339, have not been shown to affect viral fitness. At PB2 position 339 Cambodian clade 2.3.2.1c viruses predominantly contain threonine (339T). There are contradictory reports about the effect of 339T on viral replication and pathogenicity. One report showed that when in combination with 147T and 588T, 339T increased the polymerase activity and replication of H5N1 AIVs in mammalian cells and increased pathogenicity in mice [84]. Whereas, another report suggested that 339T attenuates PB2 cap binding capabilities, decreasing viral polymerase activity, replicative efficiency and decreases H5N1 AIV virulence in mice [85]. A single isolate from 2015 had a methionine substitution at this position (339M). The T339M mutation has been suggested as an A(H5N1) adaptation to humans and was shown to enhance the growth in a mammalian cell line, particularly when in combination with PB2 mutations 249G or 309D [86]. The precise effect of mutations in PB2 at position 339, particularly 339T, remains unclear.

Vaccination is an effective way to prevent influenza infection [111]. Given that there are no A(H5N1) vaccines commonly used in humans, antiviral prophylaxis and treatment is the best option for limiting A(H5N1) infection and transmission. Therefore, we screened the NA and MP genes from Cambodian A(H5N1) isolates for markers indicative of resistance to the two classes of antiviral drugs available to treat influenza infections: adamantanes (adamantine and rimantadine) and neuraminidase inhibitors (oseltamivir, zanamivir, peramivir and laninamivir). Resistance to adamantanes is widespread and has been reported in seasonal influenza viruses as well as A(H5N1) AIVs [74,112]. In this study, two A(H5N1) clade 2.3.2.1c viruses had M2 amino acid substitutions indicative of resistance to adamantanes, S31N and A30T (Table 1). Whereas none of the viruses had molecular markers indicative of resistance to NAIs. NAI sensitivity was confirmed in a subset of thirty Cambodian A(H5N1) isolates with all viruses tested being highly susceptible to all four NAIs (S5 Table).

The A(H5N1) clade switch that occurred in Cambodia in 2014 suggests that the clade 2.3.2.1c viruses may have better fitness than the clade 1.1.2 reassortant viruses in poultry. However, the decrease in human cases indicates their zoonotic potential may be reduced. The clade 1.1.2 A(H5N1) reassortant viruses caused 64% (n = 34) of the overall Cambodian A(H5N1) human cases during a period of approximately 15 months from 2013 to 2014. Comparatively, only a single human case has been documented in Cambodia as the result of infection with clade 2.3.2.1c, despite nearly 6 years of circulation in the country. A molecular analysis of the clade 1.1.2 A(H5N1) viruses performed previously showed they do not typically contain major markers associated with AIV adaptation to mammals [23]. However, four HA mutations associated with an increase in binding to human-type α2,6 receptors were conserved in the population, including: S123P, S133A, S155N, and K266R [23]. Comparatively, the Cambodian clade 2.3.2.1c A(H5N1) viruses also have the S133A and S155N substitutions in 100% and 97% of viruses, respectively (Table 1). Though they do not have the S123P and K226R substitutions, other molecular markers associated with an increase in α2,6 binding were identified that are not common to clade 1.1.2 AIVs. It is possible differences in the viral genes other than HA contribute to this phenotype. The molecular basis for the decrease in Cambodian clade 2.3.2.1c A(H5N1) transmissibility to humans, compared to the previous circulating clade 1.1.2 viruses remains unclear.

It is important to note that the majority of data available for analysis is from 2015, as limited LBM sampling was performed in 2014 and 2016. Therefore, it is likely the viral diversity in these two years was higher than was presented in this study. Analysis of the genetic diversity of the Cambodian clade 2.3.2.1c A(H5N1) viruses shows fluctuations occurred each year from 2014 to 2016 with peaks typically occurring around December or January (Fig 5). This coincides with the Cambodian dry season that has previously been documented as a period of high AIV prevalence in Cambodia [22,54]. Sampling and analysing the molecular traits of viruses in subsequent years will be crucial to track viral evolution and diversity in Cambodia. Analysing molecular markers associated with changes in viral fitness is useful to rapidly assess the biological characteristics and risk associated with AIVs. However, moving forward it is important to verify the molecular profile of the Cambodian viruses by performing further *in vitro* and *in vivo* virulence testing. Particularly as the effect of substitutions can be context dependent and novel substitutions associated with an increase in viral fitness may emerge. This will provide a more comprehensive assessment of the risk Cambodian A(H5N1) pose to the community.

Overall, in this study we genetically characterised clade 2.3.2.1c A(H5N1) viruses circulating in Cambodia from 2014 to 2016. Analysis shows that reassortment of internal genes between A(H5N1) clades and other AIV subtypes does occur in the region. Continual surveillance and characterisation of AIVs is essential to limit the impact of this disease on animals, the economy and human health.

## Supporting information

S1 Figs Bayesian maximum clade credibility (MCC) phylogenetic tree of Cambodian clade 2.3.2.1c virus a) HA and b) NA genes detected from 2014 to 2016. Cambodian viruses are coloured based on the year they were detected: viruses from 2014 are purple, 2015 are blue and 2016 are red. The single human Cambodian clade 2.3.2.1c virus is indicated by a black circle. Trees were generated with BEAST v1.84 using GTR+Γ with the SRD06 nucleotide substitution model. The tree branch lengths are time-proportional and the time scale is indicated on the x axis. The proposed tMRCA is displayed at each node.
(PDF)

S2 Figs Maximum likelihood phylogenetic trees for NA, MP and internal genomic segments of Cambodian A(H5N1) viruses detected from 2014 to 2016. a) NA, b) PB2 c) PB1 d) PA e) NP f) MP and g) NS. Trees were generated with IQ-Tree using GTR+ Γ and 1,000 ultrafast bootstrap replicates. Taxa names show viral subtype, HA clade designation and viral strain name. Cambodian viruses are coloured based on the year they were collected. Viruses detected prior to 2013 are coloured orange, viruses from 2013 are green, viruses from 2014 are purple, 2015 are blue and 2016 are red. Segment lineages are indicated on the right hand side of the tree. For NA amino acid differences relative to the closest related WHO candidate vaccine virus (A/duck/Vietnam/NCVD-1584/2012) are shown next to the phylogeny in grey. Mutations listed at branches on the left hand side of the tree prevail in descendant viruses. Mutations listed next to viral taxa on the right hand side of the tree are found in the individual virus. Underlined mutations are those that have been previously reported to affect viral virulence. Bootstrap values of 70 or greater are displayed on nodes.
(PDF)

S1 Table List of Cambodian A(H5N1) viruses detected between 2014 and 2016 that were included in this analysis with details on sample collection, AIV genotypes and sequencing accession numbers. (XLSX)

S2 Table Molecular inventory of the Cambodian A(H5N1) viruses between 2014 and 2016: a) PB2, b) PB1, c) PA, d) HA, e) NP, f) NA, g) MP, h) NS. (XLSX)

S3 Table Selection pressure analysis of the Cambodian A(H5N1) genes using FEL, FUBAR, MEME and SLAC. (XLSX)

S4 Table Predicted HA and NA N-glycosylation sites of Cambodian A(H5N1) viruses between 2014 and 2016. (XLSX)

S5 Table Sensitivity of Cambodian A(H5N1) viruses to neuraminidase inhibitors (zanamivir, oseltamivir, peramivir and laninamivir). (XLSX)
