# Supplementary figures and images for: Diversity of A(H5N1) clade 2.3.2.1c avian influenza viruses with evidence of reassortment in Cambodia, 2014-2016
Source: PLoS One. 2019 Dec 9;14(12):e0226108. doi: 10.1371/journal.pone.0226108 (PMC6901219; doi:10.1371/journal.pone.0226108)

**S1a) HA**

## Key

■ 2014

2015

2016

\* Poultry outbreak

- Human AIV

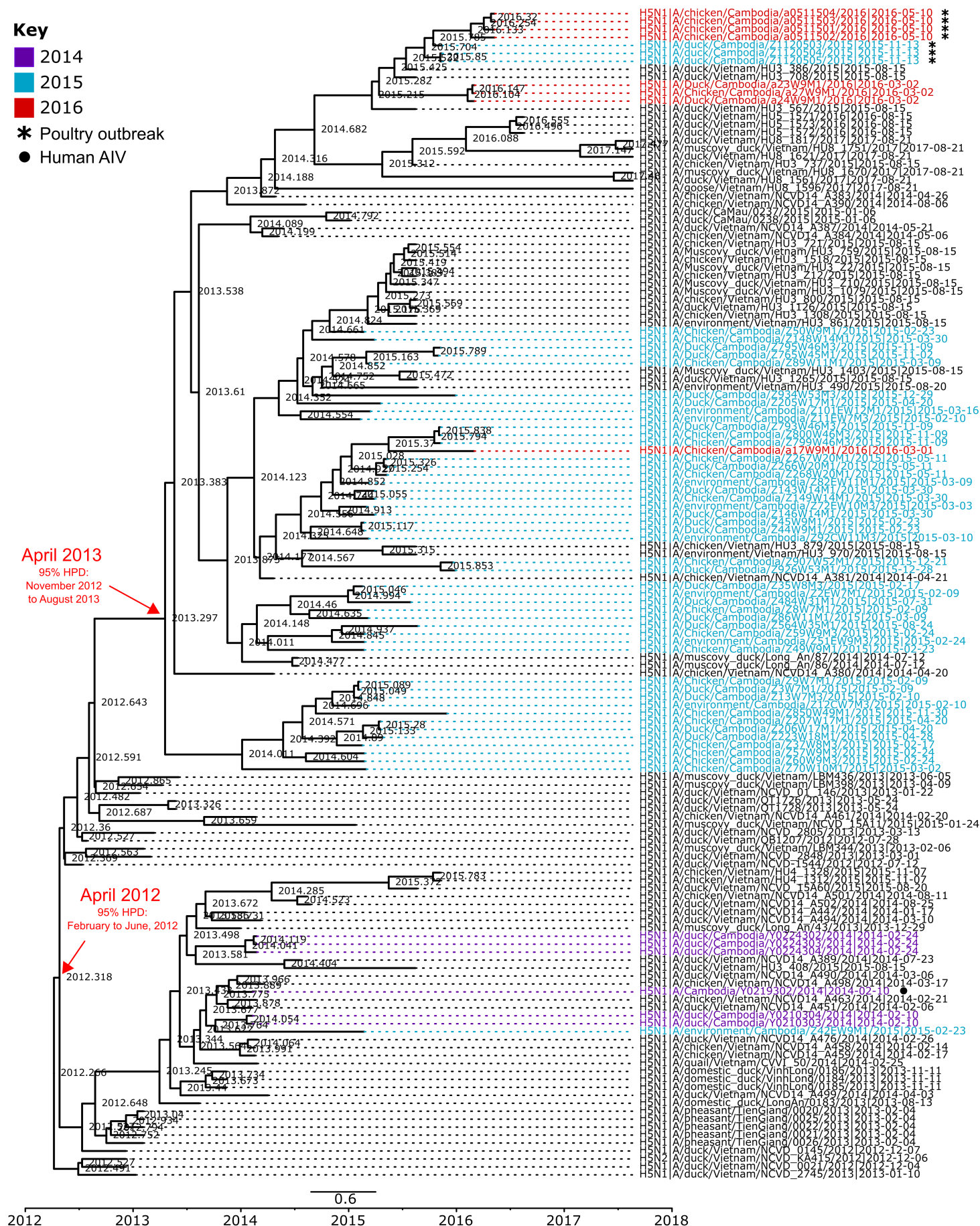

**S1b) NA**

## Key

■ 2014

2015

2016

\* Poultry outbreak

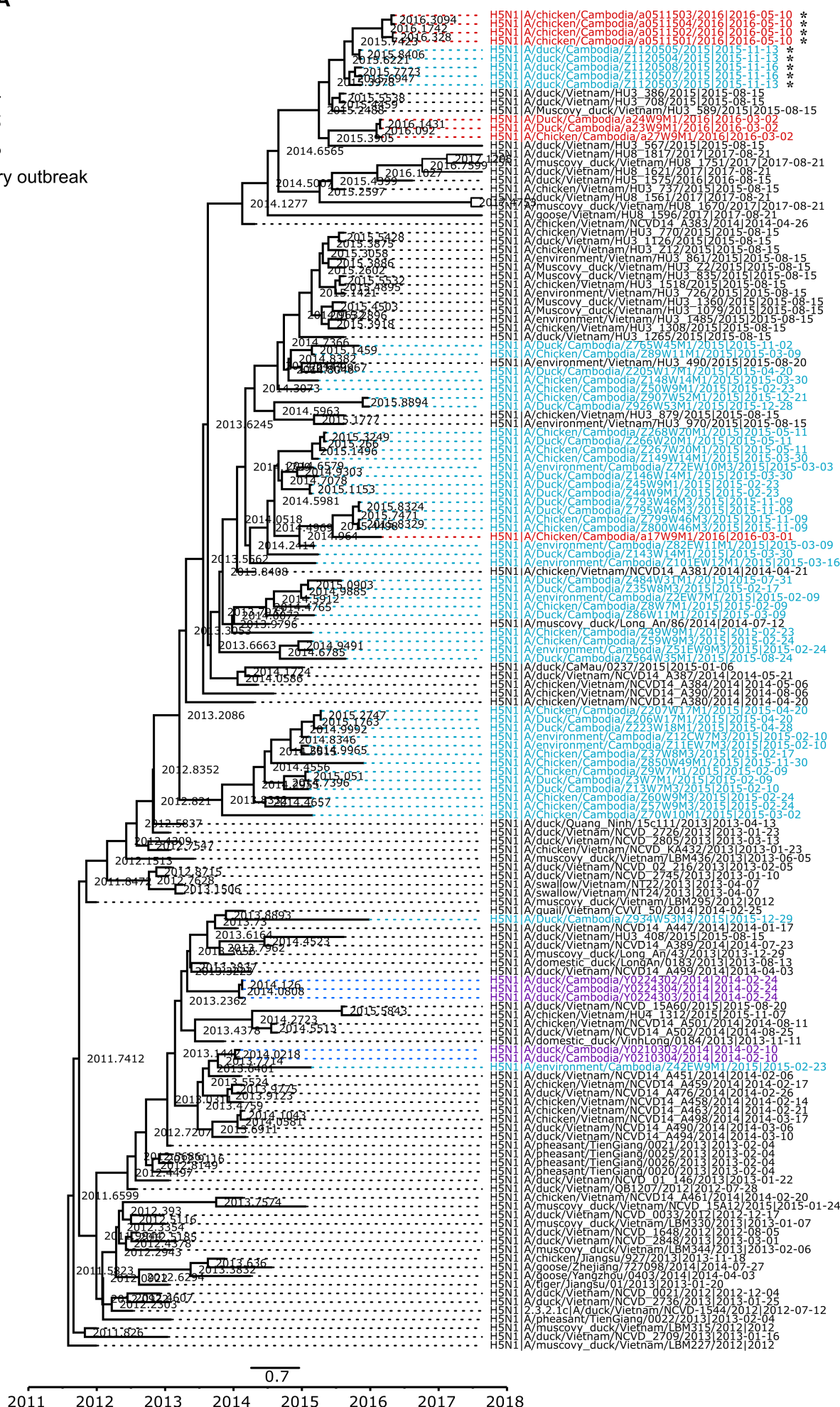

Supplement: S1 Figs — Cambodian viruses are coloured based on the year they were detected: viruses from 2014 are purple, 2015 are blue and 2016 are red. The single human Cambodian clade 2.3.2.1c virus is indicated by a black circle. Trees were generated with BEAST v1.84 using GTR+Γ with the SRD06 nucleotide substitution model. The tree branch lengths are time-proportional and the time scale is indicated on the x axis. The proposed tMRCA is displayed at each node. (PDF) [file pone.0226108.s001.pdf]

**S2a) NA**

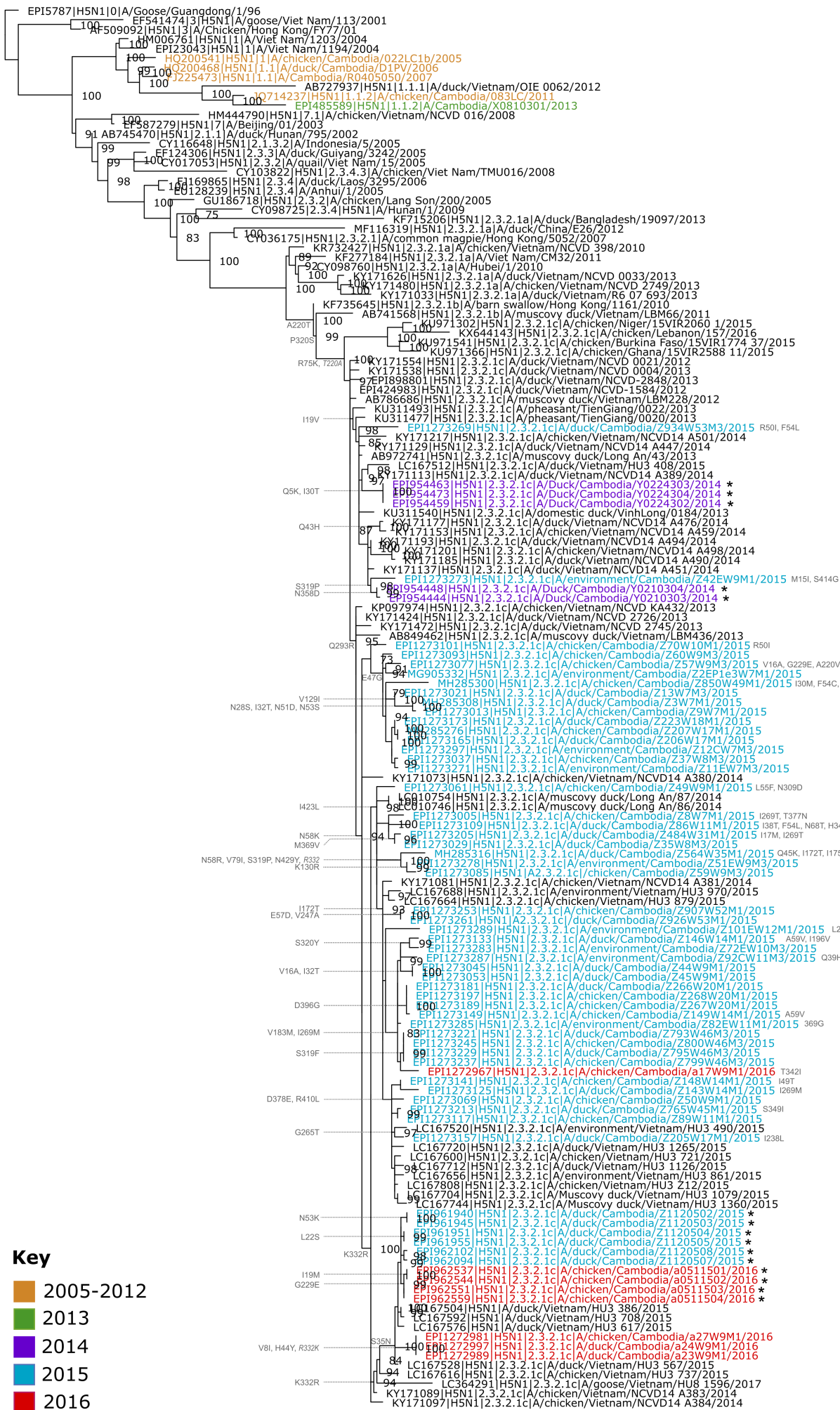

6B

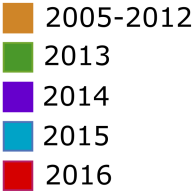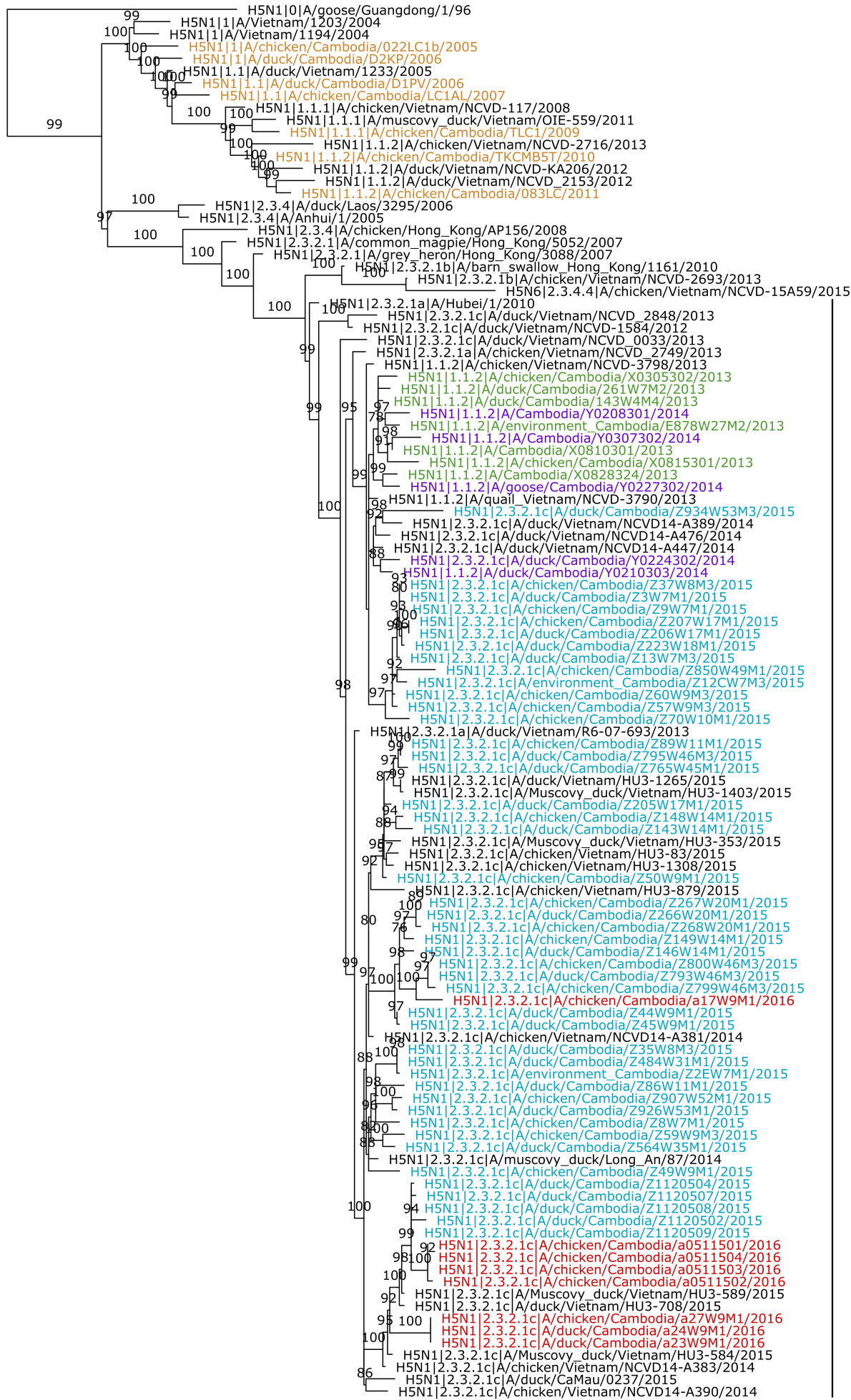

1A

- 2005-2012
- 2013
- 2014
- 2015
- 2016

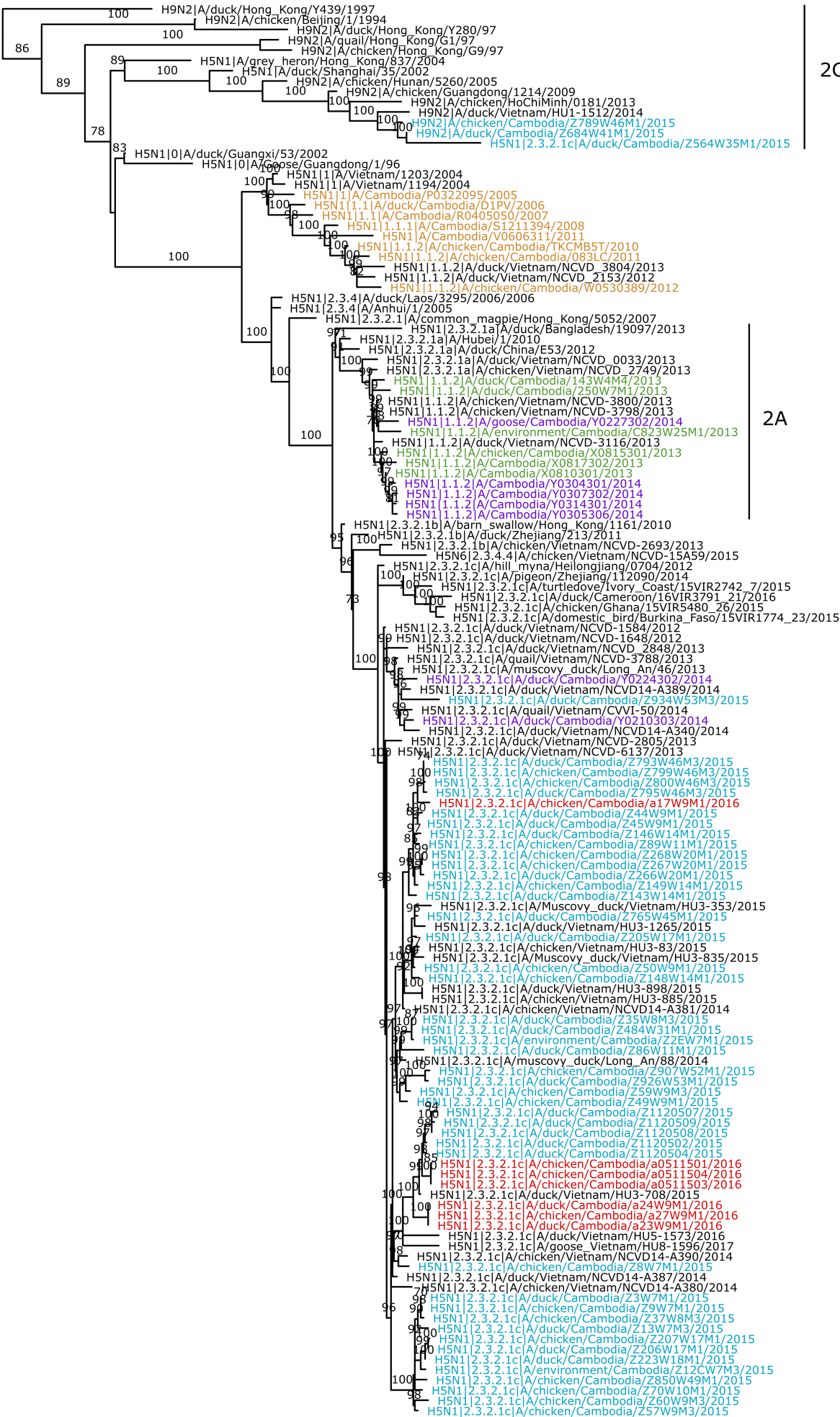

2A

2B

- 2005-2012
- 2013
- 2014
- 2015
- 2016

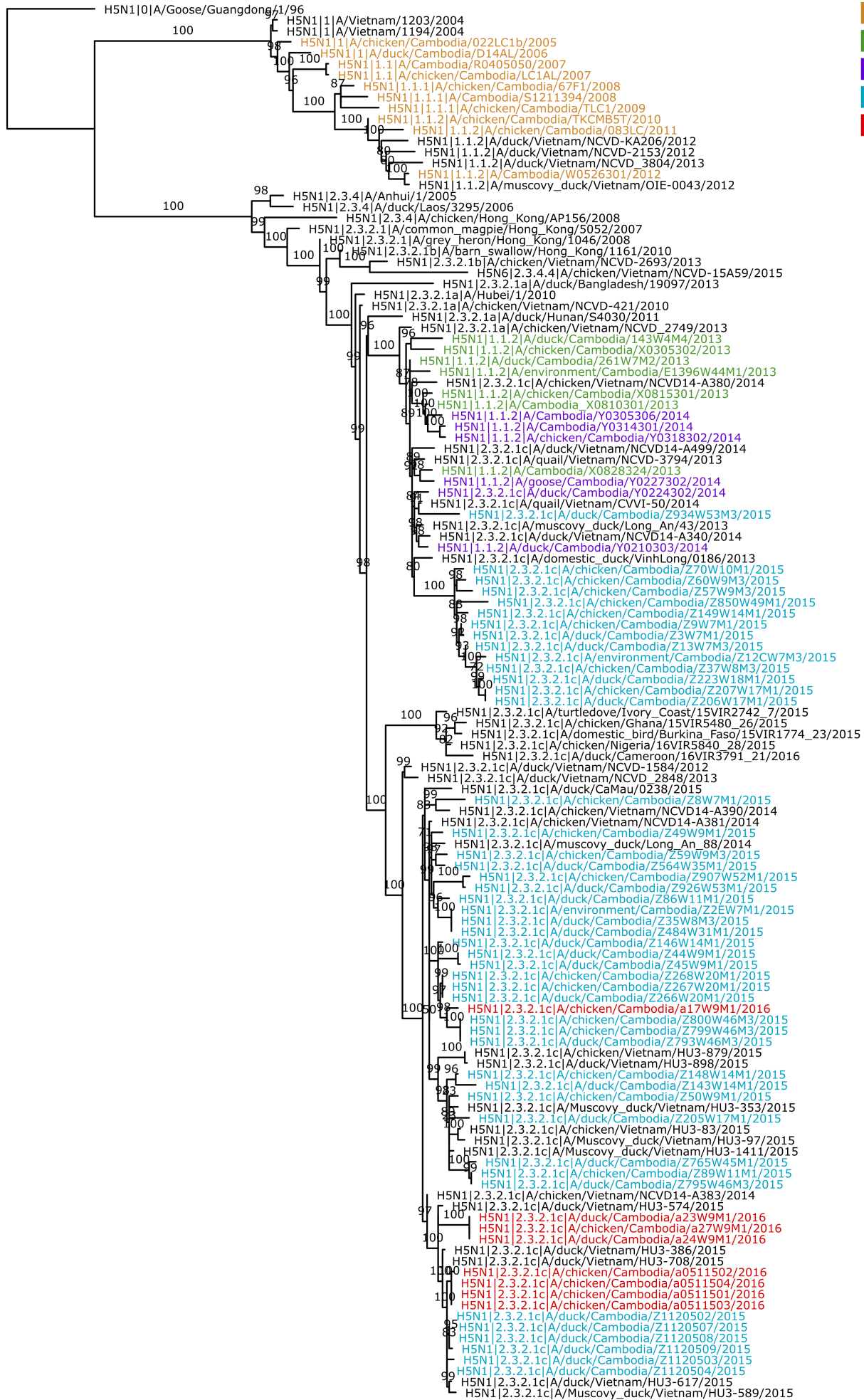

|  |
|--|
|  |
|--|

■ 2005-2012  
■ 2013  
■ 2014  
■ 2015  
■ 2016

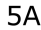

5B

S2f) MP

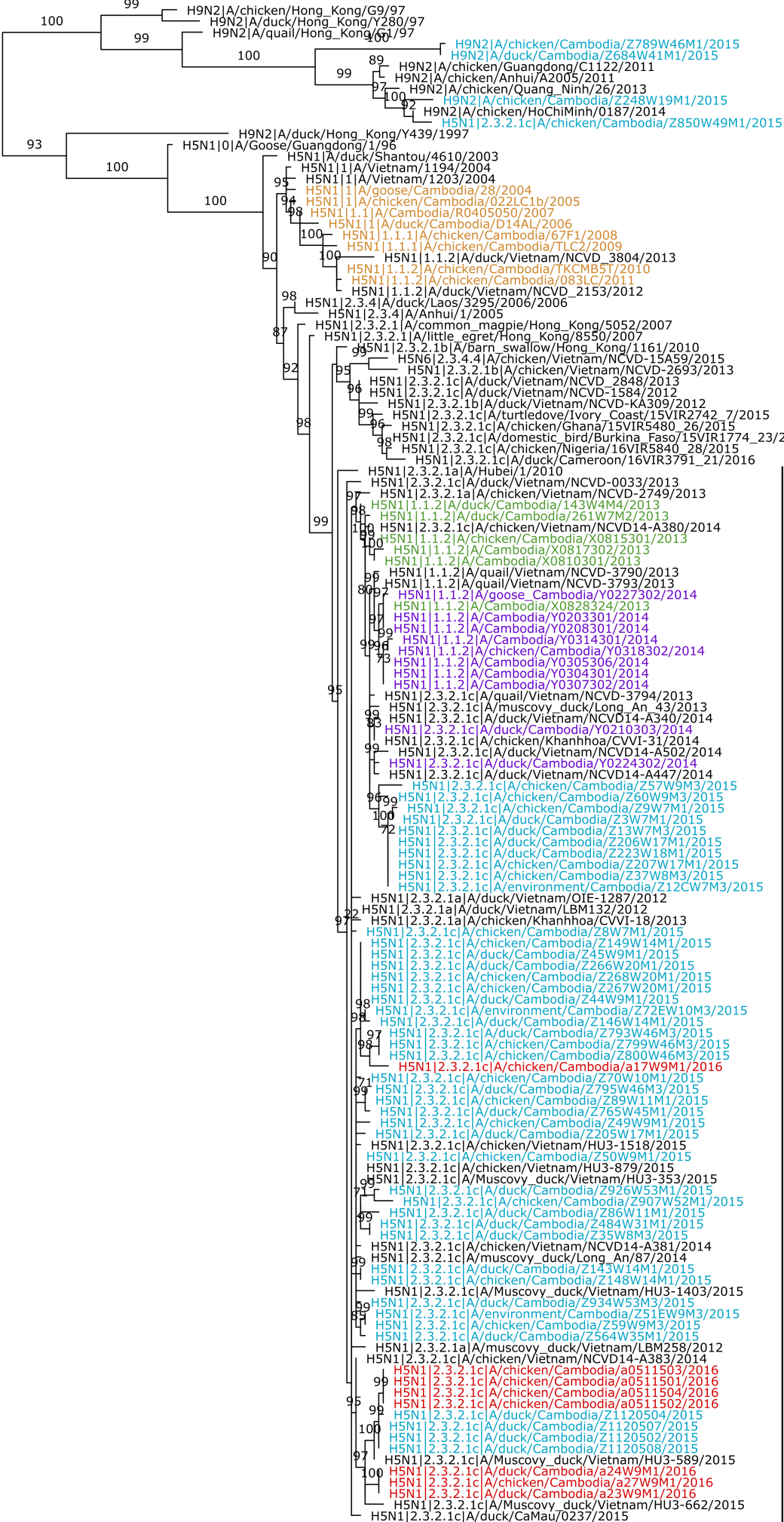

Key

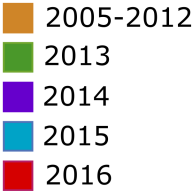

7C  
(H9  
lineage)

7B

7A

- 2005-2012
- 2013
- 2014
- 2015
- 2016

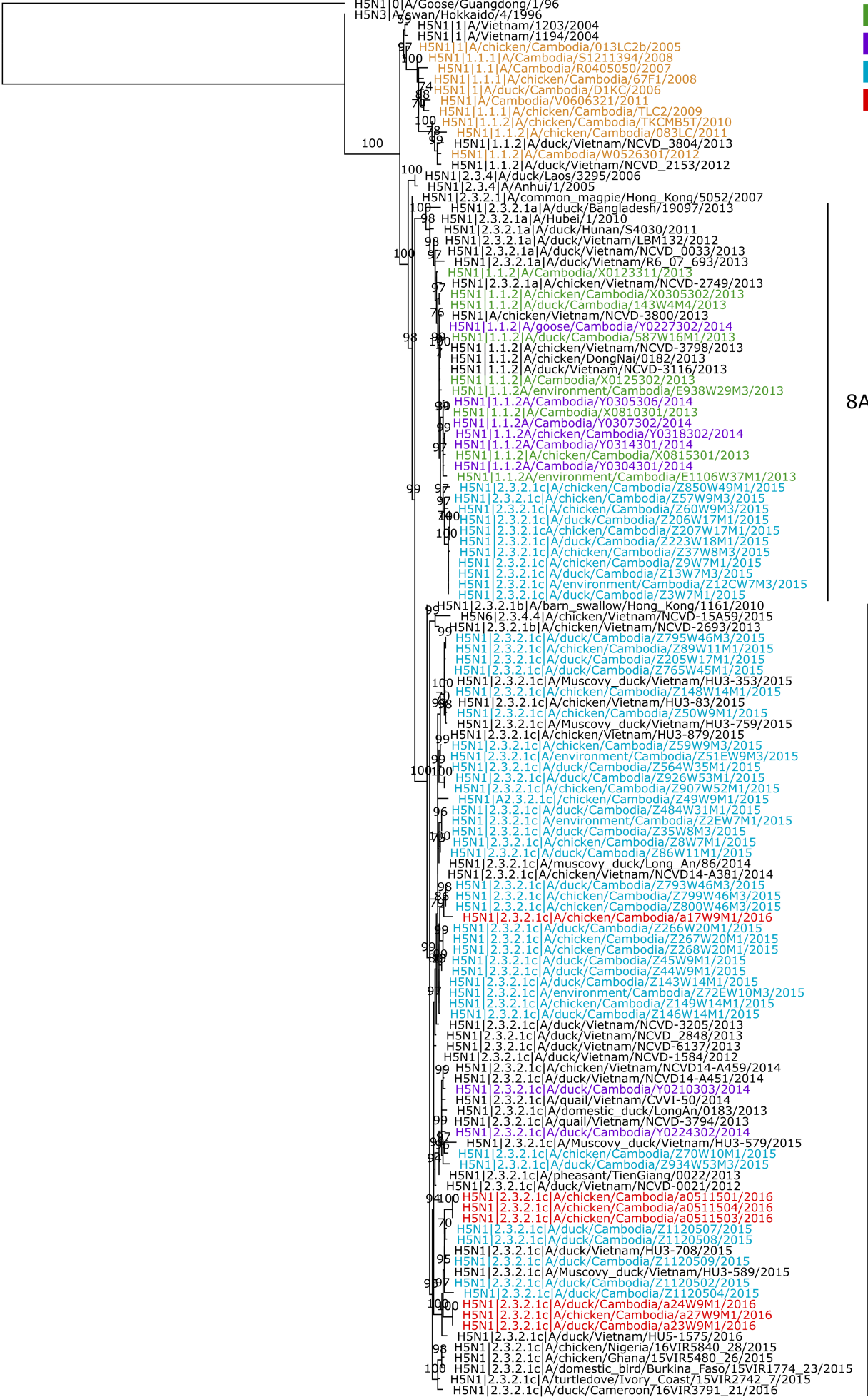

Supplement: S2 Figs — a) NA, b) PB2 c) PB1 d) PA e) NP f) MP and g) NS. Trees were generated with IQ-Tree using GTR+ Γ and 1,000 ultrafast bootstrap replicates. Taxa names show viral subtype, HA clade designation and viral strain name. Cambodian viruses are coloured based on the year they were collected. Viruses detected prior to 2013 are coloured orange, viruses from 2013 are green, viruses from 2014 are purple, 2015 are blue and 2016 are red. Segment lineages are indicated on the right hand side of the tree. For NA amino acid differences relative to the closest related WHO candidate vaccine virus (A/duck/Vietnam/NCVD-1584/2012) are shown next to the phylogeny in grey. Mutations listed at branches on the left hand side of the tree prevail in descendant viruses. Mutations listed next to viral taxa on the right hand side of the tree are found in the individual virus. Underlined mutations are those that have been previously reported to affect viral virulence. Bootstrap values of 70 or greater are displayed on nodes. (PDF) [file pone.0226108.s002.pdf]
